# Evaluation of major complications associated with percutaneous CT-guided biopsy of lung nodules below 3 cm

**DOI:** 10.3906/sag-1908-73

**Published:** 2020-04-09

**Authors:** Özgür ÇAKIR, İsa ÇAM, Ural KOÇ, Ercüment ÇİFTÇİ

**Affiliations:** 1 Department of Radiology, Kocaeli University School of Medicine, Kocaeli Turkey; 2 Ankara Gölbaşı Sehit Ahmet Özsoy State Hospital, Ankara Turkey

**Keywords:** Lung, biopsy, radiology, interventional

## Abstract

**Background/aim:**

The aim of this study was to evaluate retrospectively the incidence and risk factors for the serious complications of pneumothorax and/or parenchymal haemorrhage occurring after computed tomography (CT) guided transthoracic biopsy.

**Materials and methods:**

The relation between the incidence of pneumothorax and parenchymal haemorrhage due to biopsy, age, sex, lesion localization, lesion size, duration of the procedure, depth of lesion, number of pleural insertions of the biopsy needle and pathology results were statistically evaluated.

**Results:**

Between 2016 and 2017, 309 cases with lesions below 3 cm in diameter of a total of 768 (40.2%) CT-guided chest biopsy patients were selected for retrospective review. The rate of pneumothorax and parenchymal haemorrhage was 18.1% (59/309) and 51% (158/309), respectively post biopsy. The number of needle pleural insertions was correlated with the development of pneumothorax (P = 0.002). At regression analysis, for parenchymal haemorrhage, lesion depth (P < 0.001) and total procedure time (p=0.036) were determined as the most important independent risk factors.

**Conclusion:**

Pneumothorax and parenchymal haemorrhage are common complications after CT-guided percutaneous biopsy. The minimum number of needle-pleural insertions, the optimal access route to the lesion and as quick as possible biopsy procedure should be selected to reduce the risk of pneumothorax and parenchymal haemorrhage.

## 1. Introduction

Today, with advances in technology and widespread use of radiological examinations, pulmonary masses tend to be diagnosed earlier. Solitary pulmonary nodules are malignant in between 20% and 40% of cases although in the 55–75 years old age group this rate increases 1.2-fold for every 10 years of additional age [1].

The development and use of percutaneous transthoracic biopsy (PTB), has led to diagnostic accuracy rates of 75%–81% [2]. Other diagnostic techniques include sputum cytology, bronchoscopy, and thoracoscopy [3,4]. 

PTBs have become increasingly used due to the low risk of serious complications, repeatability, compatibility with local anaesthesia and low cost, as well as high diagnostic accuracy [5,6]. Although the risk of complications is perceived to be low, they occur nevertheless, with the most common complications of PTB being pneumothorax and parenchymal haemorrhage. In a meta-analysis, it was reported that the rate of both pneumothorax (8.4%–45.3%) and parenchymal haemorrhage (3.3%–54.5%) varied widely following lung nodule biopsy [7]. Other possible severe complications would include seeding of metastases by shedding malignant cells from the biopsy needle and haemoptysis [8,9]. Fatal complications such as pericardial tamponade and systemic air embolism have rarely been reported in the literature [8,10].

Lung nodules with size below 3 cm, considered as T1 (T for extent of the primary tumour) and those with a size of above 3 cm are considered as T2 in the 8th edition Lung Cancer Stage Classification [11]. According to this classification, T1 and T2 tumours have a 5-year life span of 92% and 68%, respectively [11]. Therefore, rapid identification and resection of the pulmonary nodule is crucial to improve prognosis and survival.

Lesion size, depth of the parenchyma and the number of pleural needle entries have been associated with pneumothorax and haemorrhage in the literature, although reports are sometimes inconsistent and contradictory [12–14].

In this retrospective study, we aimed to evaluate the relationship between pneumothorax and parenchymal bleeding complications, age, sex, lesion location, lesion depth, lesion size, duration of the procedure, number of needle pleural insertions and pathology results as a result of percutaneous transthoracic biopsy.

## 2. Material and methods

In this study, images of 768 patients, presenting to Kocaeli University Medical Faculty Hospital between 2016–2017 who were investigated with CT-guided percutaneous transthoracic biopsy because of lung nodules evident on thoracic imaging, were evaluated.

### 2.1. Inclusion criteria 

Patients with lung nodules less than 30 mm in diameter without histopathologic diagnosis were detected by computed tomography (CT) or positron emission tomography (PET).

### 2.2. Exclusion criteria

Masses over 30 mm in size (n = 220), masses located on the chest wall and mediastinum (n = 130), severe heart failure (n = 62), severe renal or hepatic insufficiency (n = 27), and patients determined to have complications such as pre-existing severe haemorrhage (n = 12) or pneumothorax (n = 8) which would have compromised the safety of the biopsy during the procedure were excluded from the study.

Lesion size, location, depth, duration of procedure, the number of sampling attempt and histopathological data were evaluated as the main factors affecting pneumothorax and the rate of haemorrhage after the biopsy procedure. Procedures were performed by 2 experienced radiologists with more than 5 years’ of experience regarding transthoracic biopsy (ÖÇ, İÇ).

The lesions were divided into 3 groups according to their largest diameter: <10 mm, 10–19 mm, 20–29 mm. The lesions were divided into 5 groups according to their location: left upper, left lower, right upper, right middle, and right lower. Benign lesions were divided into 4 groups according to pathology: organized pneumonia, hamartoma, anthracosis, and tuberculosis. Malignant lesions were also divided into four groups according to pathology: adenocarcinoma, squamous cell cancer, small cell cancer, and metastasis. The lesions were also classified according to position and divided into 3 groups: 20 mm and less, 21–40 mm, and more than 41 mm based on the shortest distance to the pleura.

Pneumothorax development was evaluated by post biopsy images. After the procedure, the development of parenchymal haemorrhage was evaluated. The haemorrhage was classified according to their longest dimension as 20 mm below, between 21–50 mm and above 51 mm. Procedure times were calculated based on the time interval between admission of the patient to CT unit and the last CT scan for examination. Biopsy needle applications (NoP) were recorded, based on each pleural entry procedure for biopsy.

For this study, verbal and written informed consent was obtained from all patients before the procedure with the approval of ethics committee.

### 2.3. Procedure

Bleeding time and full blood count were obtained from all patients. Preoperative imaging studies (CT, PET CT) were examined to determine the lesion location in the lung and the relationship of the lesion to surrounding vascular structures and organs. All biopsies were performed on a 16-section multislice CT machine (Aquilion, Toshiba, Tokyo, Japan) using a 17 G coaxial needle and an 18G fully automated tru-cut gun (Bard Monopty Disposable Core Biopsy Instrument, Bard, Tempe, Ariz). The procedures were planned as the shortest path from the skin to the lesion and the patients were biopsied in supine, prone, and oblique positions. The entry point is marked on the skin by using CT scan, without penetrating the pleura, and local anaesthesia was applied with 2% lidocaine. The distance of the needle to the pleura and other structures were evaluated and pleura was passed after puncture. After reaching the target lesion with the coaxial needle, fine needle aspiration biopsy and tru-cut biopsy were performed. Biopsy procedure was performed at least twice, taking the patient’s tolerance and the adequacy of the specimen into consideration. Samples were prepared on a microscope  slide and those taken with the tru-cut gun were put into 4% formaldehyde for fixation and sent to the pathology laboratory. 

CT images obtained after removal of the coaxial needle were used to evaluate complications. After the procedure, the patients developing small pneumothorax received oxygen-bed rest for 24 hours and they were evaluated by chest x-ray. A chest tube was used in patients with significant dyspnoea haemorrhage and pneumothorax.

### 2.4. Statistical analysis

Measurement data were expressed as mean ± standard deviation, nominal and categorical data were expressed as number (n) and frequency (%). According to the normality test result, the Mann–Whitney U test and Independent samples t-test were used for between-group comparisons. Categorical variables were compared using the chi-square test. The relationships between variables were examined using correlation analysis and Spearman correlation coefficient was calculated. The variables which were found statistically significant at correlation analysis on complication (dependent variable) were examined with multivariate binary logistic regression analyses. Value of P < 0.05 was considered statistically significant. Statistical analysis was performed using statistical package for the social sciences, version 21 (IBM Corp., Armonk, NY USA).

## 3. Results

**Figure 1:**
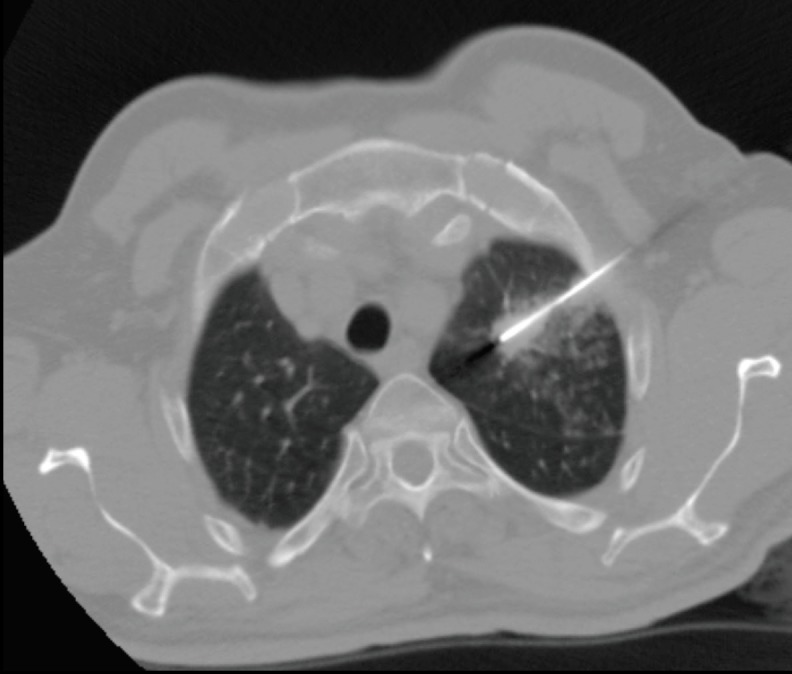
CT-guided lung biopsy of left sided 16 mm pulmonary nodule and parenchymal haemorrhage during biopsy.

**Figure 2:**
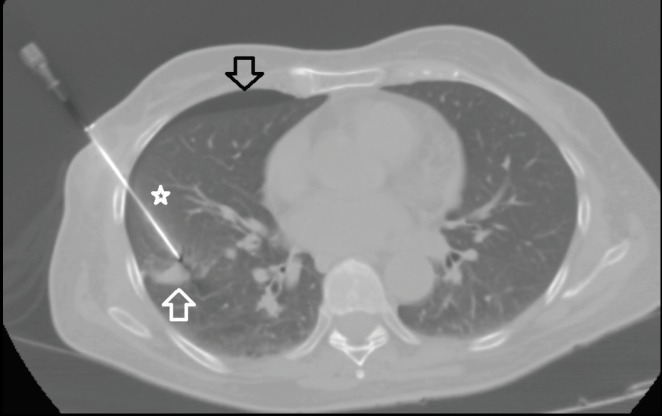
CT-guided lung biopsy of right sided 24 mm pulmonary nodule (white arrow) with tru-cut biopsy needle (star) and pneumothorax (black arrow).

Between 2016 and 2017, 768 patients who underwent percutaneous transthoracic biopsy were evaluated and 309 patients with lesions of <30 mm by largest diameter were included in the study. The fine needle and tru-cut biopsies were performed in all patients (Figure 1,2).

The mean age, standard deviation of the patients (62.6 ± 10.9 years; range, 28–87 years): 244 men (mean age, 63.1 ± 10.5 years) and 95 women (mean age, 60.5 ± 13.3 years). The mean lesion size was 19.9 ± 6.9 mm. The distribution of locations was; right upper 115 (37.2%), right middle 26 (8.4%), right lower 43 (13.9), left upper 68 (22%), left lower lobe 57 (18.4%). The mean lesion depth was 14 ± 6.5 mm. It was found that 285 (92.2%) patients required a single penetration, 20 (6.5%) required 2 penetrations, and 4 (1.3%) of the patients required 3 penetrations. Mean procedure time was determined as 20.5 ± 6.9 min (Table 1).

**Table 1:** Characteristics of the 309 study patients. Data are given as n (%) or mean ± standard deviation.

Sex		Procedure time (min)	20.5 ± 7.5
Male	244 (79%)	Lesion depth (mm)	14 ± 6.5
Female	6 (21%)	Number of punctures	
Age (years)	62.6 ± 10.9	1	285 (92.2%)
Lesion localization		2	20 (6.5%)
- Left upper lobe	68 (22%)	3	4 (1.3%)
- Left lower lobe	57 (18.4%)	Pathology results	
- Right upper lobe	115 (37.2%)	Benign	211 (68.3%)
- Right middle lobe	26 (8.4%)	- Organized pneumonia	130 (61.6%)
- Right lower lobe	43 (13.9%)	- Hamartoma	39 (18.5 %)
Lesion size (mm)	19.9 ± 6.9	- Chronic fibrosis-anthracosis	28 (13.3%)
Pneumothorax	56 (18.1%)	- Tuberculosis	14 (6.6 %)
Pneumothorax size (mm)	8 ± 5.9	Malignancy	98 (31.7%)
Haemorrhage	158 (51.1%)	- Adenocarcinoma	47 (48 %)
- None	151 (48.9%)	- Small cell lung cancer	34 (34.7%)
- Mild (0–20 mm)	113 (36.6%)	- Metastasis	11 (11.2 %)
- Moderate (20–50 mm)	41 (13.3%)	- Squamous cell lung cancer	3 (3.1%)
- Severe (50 mm-up)	4 (1.3%)	- Mesothelioma	3 (3.1%)

The lung lesions included in the study were divided by size range less than 30 mm. The mean size of the lesions was 21 mm. The distribution of the size of the lesions was: 31 (10%) between 0 and 10 mm; 122 (39.5%) between 11 and 20 mm; and 156 (50.5%) between 21 and 30 mm (Table 1).

According to the biopsy results, 211 benign (68.3%) and 98 malignant (31.7%) masses were detected. Benign masses included organized pneumonia (130, 61.6%), hamartoma (39, 18.5%), chronic fibrosis-anthracosis (28, 13.3), tuberculosis (17, 31.7). Malignant masses included adenocarcinoma (47, 48%), small cell lung cancer (34, 34.7%), metastasis (11, 11.2%), squamous cell lung cancer (3, 3.1%), and mesothelioma (3, 3.1%) (Table 1).

Pneumothorax and parenchymal hemorrhage were evaluated after biopsy. Pneumothorax was developed in 56 (18.1%) patients who underwent biopsy, and the mean thickness of pneumothorax was 8 ± 5.9 mm. Tube thoracostomy was performed in 7 patients (12.5%), and there was no patient death due to pneumothorax (Table 1).

Parenchymal haemorrhage developed in 158 (51.1%) of the patients who underwent biopsy, and no bleeding occurred in 151 (48.9%) patients. Mild bleeding (113, 36.6%), moderate bleeding (41, 13.3%), and severe bleeding (4, 1.3%) was detected (Table 1). Four patients with severe parenchymal haemorrhage and patients with moderate bleeding were discharged 1–3 days after clinical follow-up. Bronchial artery embolization was not required in any patient following bleeding. Patient death did not occur due to postoperative complications.

After PTB, factors that may affect the risk of pneumothorax and parenchymal bleeding were evaluated (Table 2). As the NoP increased there was a significant increase in the risk of pneumothorax development after transthoracic biopsy. No significant effect was found for an increased risk of pneumothorax occurrence for patient age, sex, lesion location, lesion size, procedure duration, lesion depth, and lesion pathology. 

**Table 2:** The relationship between risk factors and complications (pneumothorax and parenchymal haemorrhage) were demonstrated.

Complications	Pneumothorax		Haemorrhage		Haemorrhage size	
	r^a^	p	r^a^	p	r^a^	p
Sex	0.016	0.778	–0.099	0.082	–0.083	0.143
Age (years)	0.026	0.645	–0.058	0.306	–0.049	0.389
Lesion localization	0.005	0.932	–0.065	0.254	–0.056	0.325
Lesion size (mm)	0.030	0.595	–0.188	0.001	–0.212	<0.001
Procedure time (min)	0.036	0.527	0.225	<0.001	0.241	<0.001
Lesion depth (mm)	–0.020	0.732	0.399	<0.001	0.433	<0.001
Number of punctures (NoP)	0.345	0.002	–0.044	0.431	0.065	0.255
Pathology results	–0.004	0.940	–0.057	0.316	–0.077	0.177

Footnote: a Spearman correlation coefficient.

There was a significant difference related to lesion size, lesion depth and procedure time between parenchymal haemorrhage present or absent groups (P = 0.001, P < 0.001, P < 0.001, respectively). The risk of parenchymal haemorrhage occurrence and resulting haemorrhage size were both found to be significantly associated with lesion size, depth of lesion and longer duration of the procedure (Table 2). There was no increase in risk of haemorrhage due to gender, age, lesion localization, NoP or lesion pathology after the biopsy procedure (Table 2).

At multivariate binary logistic regression analyses, depth of lesion and procedure time were determined as an independent risk factors increasing the risk of parenchymal haemorrhage (Table 3).

**Table 3:** Multivariate binary logistic regression analysis of risk factors of haemorrhage

	B	SE	Wald	p	OR	95% CI for OR	
						Lower	Upper
Procedure time	0.039	0.019	4.400	0.036	1.040	1.003	1.079
Lesion depth	0.040	0.008	27.414	0.000	1.041	1.025	1.056
Lesion size	–0.037	0.019	3.814	0.051	0.964	0.929	1.000
Constant	–0.757	0.600	1.596	0.206	0.469		

Abbreviations: CI, confidence interval; OR, odds ratio; SE, standard error.

## 4. Discussion

The most common complication encountered in the literature following PTB is parenchymal haemorrhage with a rate of 51.1% (158/309), as in our study [7]. Most of the cases in our series experienced only mild to moderate haemorrhage and bronchial embolization was not required in any cases. In addition, there was no patient death. Pulmonary hemorrhage showed significant correlation with lesion size, lesion depth and procedure time. 

The factor of lesion depth was determined to independently increase the risk of haemorrhage (OR, 1.041; 95% CI, 1.025:1.056; P < 0.001). The relationship between pulmonary nodule depth (pathway) and pulmonary haemorrhage can be explained by the small size of the lesion (less than 3 cm by design), a large number of needle entries, a greater distance between the nodule-pleura and the proximity to the pulmonary vessels.

In our study, the relationship between nodule size and pulmonary haemorrhage is in agreement with the literature [15,16]. It has been hypothesised that small nodule biopsies are more difficult and therefore time consuming. In addition, small lesions often require multiple biopsy attempts increasing the NoP [16]. Significant correlation was also found between an increase in the duration of the procedure and haemorrhage. The factor of procedure time was determined to independently increase the risk of haemorrhage (OR, 1.040; 95% CI, 1.003:1.079; P = 0.036). Our findings also suggested that smaller size of nodule tended to prolong the procedure time and led to an increase in the risk of complications. While pulmonary haemorrhage can have serious life-threatening consequences, this was not the case in our experience. There was no need for treatment and no patient death in our study.

We hoped to find a statistically significant correlation between parenchymal haemorrhage and the number of needle pleural insertion. However, the risk of parenchymal bleeding was not correlated with an increased number of needle entries.

Pneumothorax was the second most common serious complication following PTB with a rate of 18.1% (56/309). This finding is in concordance with the literature [17]. There was a significant correlation between the number of needle entries and pneumothorax complications. Again, this is in agreement with previous reports [13,18]. We hypothesize that more needle entries lead to more defect-induced pneumothorax in the pleura during patient inspiration.

There was no statistically significant correlation between the development of pneumothorax following PTB and gender, age, lesion location, lesion size, depth, lesion pathology and procedure time. Our findings concerning lesion depth and a lack of increased risk of pneumothorax are in contrast to some previous reports [16,19].

Contrary to some other reports, there was no correlation between the development of pneumothorax following PTB performed on small lesions in our study [14,19]. However, our findings are in agreement with previous studies which reported no significant relationship between pneumothorax and lesion size [12,13]. 

This is the first study, to the best of our knowledge, to investigate if there is any correlation between histopathological diagnoses and pneumothorax and parenchymal haemorrhage after PTB. We found no significant statistical relationship between the histopathological results of the lesions biopsied and the development of pneumothorax or parenchymal haemorrhage. It was thought that the inclusion of nodules of less than 3 cm in maximum diameter and thus the exclusion of large malignant tumours during biopsy might affect the results. However, it has previously been hypothesised that smaller lesions are technically more difficult to handle and therefore would be more prone to post-biopsy complications [12,13].

There are some limitations to our study. The most important of these is the retrospective nature of the study, performed by only 2 operators. The effect of the experience of the operators, inter-operator comparison and the relationship between complication occurrence were not evaluated. In addition, the study did not examine pulmonary functions and pulmonary emphysema-bulla formations before the procedure. Pulmonary function is not routinely evaluated in our clinic and therefore there is no pre- and postbiopsy data concerning lung function. Prior to the procedure, surgery-radiotherapy-cigarette anamnesis, which could increase the risk of complications, was not taken into consideration during the assessment of data. In addition, body position (supine, prone, oblique) during the procedure, and fissure formation within the parenchyma during biopsy were not evaluated.

In conclusion, pneumothorax and parenchymal haemorrhage are the most common complications of CT-guided percutaneous lung biopsy. The data presented here suggests that the most important risk factor in the development of pneumothorax is the number of needle entries. For parenchymal haemorrhage the most important risk factors are the size of the lesion, duration of the procedure and depth of the lesion. In order to reduce the proportion of patients experiencing complications, we recommend making the duration of the procedure as short as possible, which is dependent on operator experience to some extent, planning access using shortest depth from skin to lesion and using the minimum number of pleural entries by biopsy needle to obtain adequate samples for analysis. 

Despite the limitations, the data obtained in our study may prevent unnecessary complications by suggesting how pre-procedure risk assessment may be modified by improvement in procedure planning and by giving important information to the clinician in reducing the complication risk after percutaneous transthoracic biopsy.

## Informed Consent

In this study, verbal and written informed consent was obtained from all patients before the procedure with the approval of Ethics Committee.of Kocaeli University with approval code: KÜ GOKAEK 2018/203.

## References

[ref0] (2007). A clinical model to estimate the pretest probability of lung cancer in patients with solitary pulmonary nodules. Chest.

[ref1] (1976). Precise biopsy localization by computer tomography. Radiology.

[ref2] (2012). Au Yong I. Investigating the solitary pulmonary nodule. British Medical Journal.

[ref3] (u2ca0). Early diagnosis of solitary pulmonary nodules. Journal of Thoracic Disease.

[ref4] (2002). The role of immediate cytological evaluation in CT-guided fine needle aspiration biopsies of the thorax. Diagnostic and Interventional Radiology.

[ref5] (2008). The evaluation and management of the solitary pulmonary nodule. Postgraduate Medical Journal.

[ref6] (u2c9e). Complication rates of CT-guided transthoracic lung biopsy: meta-analysis. European Radiology.

[ref7] (1976). Complications of percutaneous transthoracic needle aspiration biopsy. Acta Radiologica.

[ref8] (1992). Lung torsion after percutaneous needle biopsy of lung. American Journal of Roentgenology.

[ref9] (1989). Percutaneous transthoracic needle aspiration: a review. American Journal of Roentgenology.

[ref10] (u2c99). u2c96 u2c9b The IASLC lung cancer staging project: external validation of the revision of the TNM stage groupings in the eighth edition of the TNM classification of lung cancer. Journal of Thoracic Oncology.

[ref11] (2000). CT-guided transthoracic needle biopsy of pulmonary nodules smaller than 20 mm: results with an automated 20-gauge coaxial cutting needle. Clinical Radiology.

[ref12] (2003). CT-guided transthoracic needle aspiration biopsy of pulmonary nodules: needle size and pneumothorax rate. Radiology.

[ref13] (1999). Transthoracic needle aspiration biopsy: variables that affect risk of pneumothorax. Radiology.

[ref14] (u2c94). Risk factors for pneumothorax and bleeding after CT-guided percutaneous coaxial cutting needle biopsy of lung lesions. Journal of Vascular Interventional Radiology.

[ref15] (1999). Pneumothoraxes and chest tube placement after CT-guided transthoracic lung biopsy using a coaxial technique: incidence and risk factors. American Journal of Roentgenology.

[ref16] (u2c90). Risk factors of pneumothorax and bleeding: multivariate analysis of 660 CT-guided coaxial cutting needle lung biopsies. Chest.

[ref17] (u2c8c). CT fluoroscopy guided vs. multislice CT biopsy mode-guided lung biopsies: accuracy, complications and radiation dose. European Journal of Radiology.

